# Improving the capacity and diversity of local public health workforce to address climate impacts to health through community partnerships and problem-based learning

**DOI:** 10.3389/fpubh.2022.1090129

**Published:** 2023-01-18

**Authors:** Michael T. Schmeltz, Chandrakala Ganesh

**Affiliations:** Department of Public Health, California State University - East Bay, Hayward, CA, United States

**Keywords:** climate change, health, undergraduate, problem-based learning, workforce

## Abstract

All aspects of society are affected by our changing climate. Individuals and communities experience the health impacts associated with climate change most every day, whether or not they realize it. Increasing both the knowledge and capacity to respond to the health impacts of climate change will be imperative for future public health leaders. This manuscript will highlight three case studies in how problem-based learning was used by California State University, East Bay's Department of Public Health undergraduate students to address climate change issues for local community and government organizations. The results from problem-based learning collaborations between undergraduate students and community and government organizations have been mutually beneficial and increased the knowledge and workforce capacity of climate and health in the San Francisco Bay Area. The authors believe the use of problem-based learning is an effective model to achieve these goals. Both the students and the community benefit from these experiences and results of projects that enhance an organization's ability to prepare for and respond to climate change in their communities.

## Introduction

Global climate change continues to have significant impacts on human health. All aspects of society are affected by our changing climate (1). Individuals and communities experience the health impacts associated with climate change in their everyday lives ranging from direct exposure to extreme heat events, to rising food prices due to droughts in agricultural regions. Although the connection between climate and health has been studied and documented extensively, there continues to be a disconnect among individuals about how climate change impacts people directly in the here and now (2, 3). There has been little coordination among institutions of higher learning and development of programs to address this need (4). Only recently, formalized curriculum and programs have been established, particularly in schools and programs of public health (5–7). Many of the significant advancements in medical school education around the topic of climate and health have been advocated and requested by medical students (8, 9). However, these efforts have been quite sporadic and limited for undergraduate students and their education (10). Given the continued threat of climate change on society, particularly on human health, it will be imperative that not only clinicians but all health professionals be educated on this topic. Improvements in climate literacy, particularly when it comes to climate change impacts to health, are needed among both the larger population and public health professionals (11). One important way of improving climate literacy is through our formal education systems: K-12, undergraduate, and graduate programs. In particular, emphasis should be placed on climate and health education among pre-professionals in healthcare, public health, and other allied health fields due to the lasting impacts of changing climate on the health of individuals. Increasing both the knowledge and capacity to respond to the health impacts of climate change will be imperative for future public health leaders. Substantive investment in basic climate knowledge and climate change impacts to human health will be needed to advance our preparation for and response to climate hazards. Next to clinicians, public health professionals provide trusted voices and essential roles in combating the negative health impacts of climate change (12). To achieve this, it will be important to expand climate and health education into institutions of higher education, particularly at the undergraduate level where a significant portion of public health and allied health professionals obtain their education (13). It is also important to realize that this expansion cannot happen overnight. These changes may require new degree pathways, curricula, or pedagogical methods to inform and reinforce knowledge for climate and health education.

It is of utmost importance to integrate climate and health education into course and program learning objectives. In addition, rather than having a standalone course that provides a broad overview of climate change, engaging with climate and health related content in courses that pertain to social determinants of health, epidemiological methods, or health communication promotes deeper understanding of the specific ways that climate change influences health. Traditionally, undergraduate education has been centered on content based lectures from instructor to students, with assessment *via* quizzes and tests and a greater emphasis on knowledge assimilation and recall. Students often have a few discussion based courses where the exchange and clarification of content involving critical analysis and thinking occurs between instructor and students (14) in the classroom or *via* reflective essays and assignments. However, outside of internships or service-learning courses (i.e., courses that have an academic component in the classroom and an applied learning component outside the classroom), undergraduate students are likely to have participated in predominantly classroom learning during their college education.

To address the complex issues associated with climate change and to better prepare future leaders a more “hands-on” approach is needed to advance critical thinking and problem solving for issues related to climate change. One approach to increasing knowledge and action is through problem-based learning (PBL), which allows students to engage in “real-world” problem-solving opportunities through collaborative work with mentors and stakeholders which can be practiced in the classroom or through hands-on approaches in community settings (15). PBL has been shown to increase learning outcomes and long-term knowledge retention compared to traditional pedagogical methods (16). PBL has been used most often in medical education and law school with only a few examples of successful implementation in undergraduate public health education. Students and practitioners of public health have an opportunity to get involved in issues that impact local communities by engaging in PBL that provides applicable skills and knowledge to better address complex health issues associated with climate impacts. This manuscript will highlight three case studies in how hands-on problem-based learning was used by Cal State East Bay's Department of Public Health undergraduate students to address climate change issues for local community and government organizations.

## Context

Cal State East Bay is one of the most racially and ethnically diverse campuses in the US. It is also designated a Hispanic Serving Institution and Asian Americans and Native American Pacific Islanders Serving Institution. The Department of Public Health's student body reflects the University's student body as well as that of the surrounding community. The Department is committed to social justice and it values engagement and building collaborative partnerships between faculty, students, and community organizations. The climate crisis is of particular concern for vulnerable communities who are likely to bear the greatest burden. The Public Health capstone course in the Department of Public Health at Cal State East Bay, which is also the culminating course in the Problem Based Learning 3-course series, provides students with opportunities to work closely with community partners. In this culminating experience, leaders from community organizations identify a substantial health challenge or issue they are facing and work collaboratively with course instructors to formulate problems that students, in teams of six, advance solutions toward using their cumulative knowledge and lived experiences. This experience helps students integrate their public health knowledge and build on their critical thinking, problem solving, and team building skills. The Department of Public Health has partnered with over 15 organizations over the past 4 years and provided capacity, services, and knowledge to the community and public health organizations all while furthering the education and skills for undergraduate public health students. Some examples of projects include increasing awareness and capacity in the local health department for LGBTQ+ family planning, developing outreach and communication plans to get non-native English speakers vaccinated against COVID-19, and improving community knowledge on the benefits of urban agriculture and increased access to health foods in urban food deserts, etc. Increasingly though, climate change has been at the forefront of topics that organizations want to address, but have traditionally lacked the knowledge and/or capacity to achieve their desired outcomes. Faculty in the Department of Public Health has used this opportunity to engage with community partners to integrate climate and health issues in the capstone class. Three projects completed in the last year are detailed below.

## Details: Community case studies on climate and health education

### Case study—City of Oakland emergency services

The Department of Public Health at Cal State East Bay and the Communities of Oakland Respond to Emergencies (CORE) program, run by the City of Oakland in California have had a standing partnership for educating students at Cal State East Bay on emergency preparedness. The CORE program is a locally organized version of the Federal Emergency Management Agency's (FEMA's) Community Emergency Response Team (CERT) program. In the spring semester of 2019, CORE partnered with students from the Department of Public Health's capstone course. The students were tasked with adding workforce capacity and subject matter expertise to support CORE's program for Oakland. For background, the CORE program was partnering with the local Red Cross to assess the state of Oakland's emergency shelter system. This was in response to the devastating 2018 wildfire season, which included the deadly Camp Fire where 85 people lost their lives and was the deadliest wildfire season in recent California history (17). Because the California wildfire season is getting longer and the frequency, size, and intensity of wildfires are increasing, emergency preparedness programs like CORE need to be ready to house evacuated and displaced populations. The CORE program relies on volunteer relief organizations like the Red Cross to facilitate and support evacuation centers, though CORE manages the administrative process of securing buildings to use as evacuation shelters.

Students used the National Shelter System database (NSS) to identify shelter locations, performed site visits, and used criteria in the Shelter Facility Survey developed by the Red Cross to identify deficiencies and areas for improvement needed for the shelter to house communities impacted by hazards and disasters. Many of these shelters had not been inspected for over a decade and most did not meet the criteria outlined in the Shelter Facility Survey. With this data students were tasked with assessing the specific needs of the population of Oakland through analysis of census data and other socioeconomic indicators such as disability status, homelessness, and medical vulnerabilities. This analysis was combined with a geospatial analysis of high risk locations associated with climate hazards, such as wildfires and flooding events, and the specific locations of shelters to assess needs of the community.

Students developed a recommendation report from the data gathered from the survey, the analysis of secondary data from socioeconomic indicators, along with the geospatial analysis. The students then presented this report which identified recommendations on increasing shelter capacity, accessibility, and safety and improving dedicated funding to shelter systems to the CORE program and the Red Cross. This product provided guidance to CORE and the Red Cross to develop actionable tasks to ultimately address improvements. Two students who worked on this capstone project were hired as contractors soon after the course was completed to work on these shelter improvement projects.

### Case study—Community collaboration and climate vulnerabilities

The Department of Public Health at Cal State East Bay worked with the University of California San Francisco (UCSF) and community groups in the Bayview-Hunters Point (BVHP) neighborhood of San Francisco. UCSF is a large medical university that does not have undergraduate programs. Most academic programs at UCSF are clinically based with some Masters programs in Public Health. UCSF is also situated at the border of the BVHP neighborhood. This community carries most of the burden of San Francisco's pollution. Located in southeast San Francisco, residents are surrounded by considerable environmental threats, including a superfund site known as the US Navy's Hunters Point Naval Shipyard. In the spring semester of 2022, UCSF and BVHP formed a working group to leverage resources in hopes of addressing challenges that the neighborhood has been facing. Students from Cal State East Bay's Department of Public Health capstone course were enlisted to provide capacity and subject matter expertise to initiate projects and lay the foundation for collaborations that were needed to address environmental and climate issues the community was interested in addressing.

The first task of the project was centered on recommendations on how to bring UCSF, BVHP, and the City of San Francisco together to address community concerns since each group had been working on their own within this neighborhood. It was felt by residents that no one was listening to them and that UCSF and the City of San Francisco had their own agenda for the neighborhood without thought of community members, particularly around land use and urban development. Additionally, there were trust issues between the community and UCSF and how environmental clean-up operations were going because of incidents of falsified environmental reports and conflicts of interest among subject matter experts from UCSF (18, 19). Similar recommendations were needed to build an effective community advisory council at the city level. While there was an appointed advisory council, there was limited communication between community members and the advisory council.

The second task was to assess the community vulnerability to climate hazards. Bayview-Hunters Point has a long history of environmental pollution and community groups have been concerned about how current and impending climate hazards such as heat waves and sea-level rise would impact these polluted sites and the residents. Using a vulnerability assessment framework, students developed a report on the current and future climate impacts to BVHP. In order to successfully use this information, the other projects focused on communication and outreach plans for BVHP to engage with government officials and UCSF to initiate discussions on how to build climate resilience within the community. A plan was developed on a more equitable community advisory council between the main stakeholders in the BVHP community, the City of San Francisco and other government agencies that had oversight into climate adaptation and resiliency in this location. These products and plans facilitated community engagement on climate adaptation and resiliency planning within the BVHP community. It allowed the community to have additional resources and evidence to advocate for these changes. This opportunity provided UCSF with a community-based view point on how collaborations and partnerships can work among the BVHP community.

### Case study—City of Hayward environmental justice and climate action plan

In the spring semester of 2022, the City of Hayward was in the process of updating components of its General Plan, a planning document that provides a city or county with a policy framework to guide decision-making related to land use, growth and development, safety, and open space conservation. In particular, one of the updates was the creation of an Environmental Justice Element, which is a set of goals and policies that addresses health risks in disadvantaged communities and prioritizes improvements to achieve more equitable engagement with better health outcomes. One of the goals of the City of Hayward was to engage with community members to identify problems that have previously been left unaddressed. Working in the intersection of Environmental Justice and Public Health, Cal State East Bay students were tasked with helping the City of Hayward on four focus areas: (1) Access to clean air, (2) Access to healthy foods, (3) Access to safe and sanitary housing; and (4) Access to physical activity and recreation. Each of these projects is described in greater detail below.

One student team completed basic research to have a fundamental understanding of the problem associated with pollution burden. In 2021, Alameda County did not meet state standards for air pollutants PM2.5 and Ozone. As with many cities in Alameda County, the city of Hayward has a relatively high amount of PM2.5 in relation to other California census tracts. The student team explored the CalEnviroScreen tool to visualize the impact of traffic and pollution on the City of Hayward. They also created a survey on resident's knowledge about air pollution and administered it to residents, and incorporated the results to create policy recommendations for the City of Hayward.

The second team looked into “Access to healthy food” as a key determinant of positive health outcomes and adequate quality of life. Historically, low-income communities and communities of color have been disproportionately impacted by lack of access to healthier foods. After initial research on food access and food insecurity, the student group used the Policy Map tool to visualize SNAP retail locations and percentage of families receiving food stamps/SNAP benefits in Hayward. They created a campaign to raise awareness about the CalFresh/EBT/Pandemic EBT (electronic benefits) programs available at the Hayward Farmer's Market and surveyed residents about their access to nutritious food choices. Using these results, the team submitted policy recommendations for the City of Hayward.

The quality of housing in a community has a direct health impact on the people who reside within those homes. Low-income households disproportionately experience severe housing problems. These housing problems include physical defects to a unit, overcrowded conditions, and housing cost burden. In Hayward, ~80 percent of extremely low-income and 75 percent of very low-income households had one or more housing problems. The third student team working on this topic surveyed residents in Hayward about their living situation. They created a campaign to raise awareness about existing rebate programs for Hayward residents to create healthier homes and lower their energy bills. The student team recommended policies that might improve Hayward residents' access to safe and sanitary housing after researching existing policies that have been brought to City of Hayward City Council and reviewing policy measures that have been successfully implemented in other jurisdictions.

Access to public facilities and resources is a critical environmental determinant of health. According to the California Department of Parks and Recreation's Park Access Tool, 76 percent of residents in Hayward live in areas with < 3 acres of parks or open space per 1,000 residents. This indicates that 76 percent of residents in Hayward live in underserved areas for park access. The fourth student team created a resource matrix of services, programs, and offerings including parks, recreation centers, community programs, walking/hiking trails, bike paths, and other active transportation. They created a survey to understand how residents utilize the current services, programs, and offerings, as well as what they wish were different and any suggestions they may have. Based on the resource matrix and survey, the group provided policy recommendations to the City of Hayward to improve access to physical activity and recreation.

## Discussion

The results from problem-based learning and hands-on collaborations between undergraduate students and community and government organizations have been mutually beneficial and increased the knowledge and workforce capacity of climate and health work in the San Francisco Bay Area. This benefits students by giving them an opportunity to apply their topical knowledge to practical public health issues related to climate change. It also expands student knowledge on the implications that climate change will have on human health and the systemic issues this will likely cause to our public health and health care systems. By having climate and health program learning outcomes for undergraduates in Public Health majors, it helps to increase knowledge and workforce capacity to address current and future climate and health issues. In the case studies discussed, the immediate result for students has been either a paid internship or full-time employment opportunity for students to transition into the public health workforce, continuing or advancing projects on climate and health.

Partner organizations in these case studies were unable to initiate or complete projects because of the lack of knowledge, workers, or other resources. As climate hazards increase in frequency and intensity, local government and community-based organizations that serve impacted communities need to improve adaptive and resiliency measures. We know from past studies that providing public health services centered on climate change have been lacking. A survey from the National Association of County and City Health Officials (NACCHO) indicated that 80% of local health department directors said climate change is impacting their work but they lack expertise in their agency to respond and that the lack of resources prevented effecting programming in this area (20). Job postings mentioning “climate change” and “public health” have been increasing (21), though the question to ask is have we prepared our public health students and workforce to meet this demand?

While 2022 brought about advances in both Federal funding, through the Build Back Better Act and Infrastructure Investment and Jobs Act, and a number of sweeping climate measures in the state of California, knowledgeable workers will be needed to help fulfill these commitments to climate adaptation, mitigation, and resiliency. In the case studies discussed, the partnerships between Cal State East Bay's Department of Public Health, and local government and community organizations exemplify how knowledge and capacity from undergraduate students in Public Health were able to fulfill the needs of partner organizations on the topic of climate and health. The results of these projects have allowed partner organizations to either completely achieve their goals or move them closer to meeting these goals.

The authors believe that the use of problem-based learning, as a practical application, and community partnerships that contribute to improving the capacity and knowledge that address health threats associated with climate change is a key factor in preparing future public health leadership to equip communities for climate change. Additionally, having climate and health learning objectives in undergraduate programs will help to sustain student knowledge as they progress through their programs and majors. For the Department of Public Health at Cal State East Bay, this is made possible by dedicated and strong leadership for climate and health education among the faculty. Most public health and health-related undergraduate majors do not have requirements through accreditation bodies for climate and health topics, and recent assessments of courses and programs on climate change and health education are for graduate programs (22, 23). Though organizations like APHA and Columbia University's Global Consortium of Climate and Health Education have called for such and developed their own competencies. As noted above, there has been substantive engagement from medical students to include climate and health topics in medical school education, though there has not been an organized approach for public health, particularly at the undergraduate level. While there is an accrediting body for undergraduate and graduate public health programs, the Council on Education for Public Health (CEPH), curriculum and competencies do not require the topic of climate change to be addressed as CEPH allows programs the flexibility of having different topical material, like climate change and health, be introduced into skills-based competencies (24).

The Department of Public Health at Cal State East Bay has been successful in building partnerships with local government agencies and community organizations. This foundation has strengthened the problem-based learning curriculum used for climate and health education and can be a model for other public health programs. The program is still evolving and refining its approach is not without its challenges. First, as with other undergraduate programs, students are still building expertise in their areas of study. The authors found that students would benefit from more topical content on climate and health to help navigate them through the research portion of the problem-based learning projects. Adding required readings and resources can help with the process and add more clarity and knowledge for students. Second, clear expectations on the deliverables are needed, facilitated by the partner organization. When final deliverables were not clearly structured with specific goals and milestones, students had difficulty developing final products and reaching conclusions. Third, problem-based learning can be resource intensive in a large undergraduate program, as a low student-faculty ratio is needed to successfully guide students in their group projects. Similarly, problem-based learning is not often used at the undergraduate level and devoting time and resources to help students become comfortable with this pedagogical method is resource intensive.

As climate change continues to impact the health of individuals and communities, effective preparedness and responses will be needed to protect human health. The authors believe that integrating climate and health education in the undergraduate education of Public Health majors is a key factor for increasing both community and workforce knowledge on these topics. Additionally, the authors believe the use of problem-based learning is an effective model to achieve these goals. Both the students and the community benefit from these experiences and results of projects that enhance an organization's ability to prepare for and respond to climate change in their communities. The authors acknowledge that this requires strong leadership and the necessary knowledge of the faculty and university, but the benefits to increasing the public health workforce with climate and health educated workers will be substantially beneficial given our current and future outlook on local and global impacts of climate change.

## Data availability statement

The original contributions presented in the study are included in the article/supplementary material, further inquiries can be directed to the corresponding author.

## Author contributions

All authors listed have made a substantial, direct, and intellectual contribution to the work and approved it for publication.
